# Emergency percutaneous nephrostomy versus emergency percutaneous nephrolithotomy in patients with sepsis associated with large uretero-pelvic junction stone impaction: a randomized controlled trial

**DOI:** 10.1590/S1677-5538.IBJU.2015.0643

**Published:** 2017

**Authors:** Chi-Sen Hsu, Chung-Jing Wang, Chien-Hsing Chang, Po-Chao Tsai, Hung-Wen Chen, Yi-Chun Su

**Affiliations:** 1 Department of Infection, Saint Martin De Porres Hospital, Chiayi, Taiwan, R.O.C.;; 2 Division of Urology, Department of Surgery, Saint Martin De Porres Hospital, Chiayi, Taiwan, R.O.C.;; 3 Department of Emergency, Saint Martin De Porres Hospital, Chiayi, Taiwan, R.O.C.

**Keywords:** Sepsis, Nephrostomy, Percutaneous

## Abstract

**Introduction:**

A randomized trial was conducted prospectively to evaluate the efficacy, related complications, and convalescence of emergency percutaneous nephrolithotomy compared to percutaneous nephrostomy for decompression of the collecting system in cases of sepsis associated with large uretero-pelvic junction stone impaction.

**Materials and Methods:**

The inclusion criteria included a WBC count of 10.000/mm^3^ or more and/or a temperature of 38°C or higher. Besides, all enrolled patients should maintain stable hemodynamic status and proper organ perfusions. A total of 113 patients with large, obstructive uretero-pelvic junction stones and clinical signs of sepsis completed the study protocol. Of those, 56 patients were placed in the emergency percutaneous nephrostomy group, while the other 57 patients were part of the percutaneous nephrolithotomy group. The primary end point was the time until normalization of white blood cells (WBC) at a count of 10.000/mm^3^ or less, and a temperature of 37.4°C or lower. The secondary end points included the comparison of analgesic consumption, length of stay, and related complications. Statistical analysis was performed using SPSS® version 14.0.1. The Mann-Whitney U test, chi-square test, and Fisher’s exact test were used as appropriate.

**Results:**

The length of hospital stays (in days) was 10.09±3.43 for the emergency percutaneous nephrostomy group and 8.18±2.72 for the percutaneous nephrolithotomy group. This set of data noted a significant difference between groups. There was no difference between groups in regard to white blood cell count (in mm^3^), time to normalization of white blood cell count (in days), body temperature (in ºC), time to normalization of body temperature (in days), C-reactive proteins (in mg/dL), time taken for C-reactive proteins to decrease over 25% (in days), procalcitonin (in ng/mL), or complication rates.

**Conclusions:**

This study confirms that emergency percutaneous nephrolithotomy may be as safe as early percutaneous nephrolithotomy in a selected low risk patients with sepsis-associated large, obstructive stone.

## INTRODUCTION

Although urolithiasis is one of the most common urological diseases, it can be lethal when a urinary tract infection associated with obstructive uropathy due to upper urinary tract calculi results in bacteremia and sepsis (1). The efficacy of percutaneous nephrostomy and retrograde ureteral catheterization in decompressing the collecting system has been firmly established (2, 3). Furthermore, the high success and low complication rates of these drainage procedures have made both alternatives attractive to radiologists and urologists. Percutaneous nephrolithotomy (PCNL) remains the important contraindication for large renal calculi with untreated urinary tract infections (UTI) (4). Antegrade lithotripsy is generally not advocated for patients who are severely ill (5). However, advances in endoscopic instruments and techniques and surgeon’s familiarity with the procedure have significantly shortened operation times and increased the success rate. A randomized trial was conducted with its focus being evaluation of the efficacy, related complications, and convalescence of emergency percutaneous nephrolithotomy compared to percutaneous nephrostomy for decompression of the collecting system in cases of sepsis associated with large uretero-pelvic junction stone impaction.

## MATERIALS AND METHODS

The study was approved (STM No. 06B-008) and its related work was undertaken in Chia-Yi city and overseen by our Institutional Review Board at St. Martin De Porres Hospital. All procedures performed in studies involving human participants were in accordance with the ethical standards of the institutional and/or national research committee and in compliance with the 1964 Helsinki declaration and its later amendments or comparable ethical standards. All patients were asked to sign an informed consent form before granting their participation. The study was designed to be a randomized, controlled trial and was carried out from January, 2007 to July, 2013. A sample size of 45 patients was required in order to detect a 30% difference in the proportions of the trial parameters (e.g. complication rates, such as time until WBC normalization at 10.000/mm^3^ or less and a temperature of 37.4°C or lower, length of stay) in the treatment groups at a significance level of 0.05 and a power of 80%. Adult patients admitted to the emergency room or to the hospital with large (>20mm), obstructive uretero-pelvic junction stones and clinical signs of sepsis were asked to participate in this randomized study. The inclusion criteria included a WBC count of 10.000/mm^3^ or more and/or a temperature of 38ºC or higher. Besides, all enrolled patients should maintain stable hemodynamic status and proper organ perfusions. Patients were excluded from the study if they had uncorrected coagulopathy, urinary diversion, pregnancy, a solitary kidney, severe sepsis, septic shock, and an unwillingness or were otherwise unable to commit to the study’s follow-up protocol.

Preoperative and admission-related data included urinalysis, urine culture, blood culture, complete blood count, biochemistry study, renal ultrasound, plain kidney-ureter-bladder X-film, intravenous urography, and whole abdominal computed tomography (CT) were obtained and evaluated upon admission. Intraoperative findings, stone composition, and outcome were also recorded. Stone length was calculated according to the longest diameter, and the stone burden was calculated by multiplying its length by its width. The stone-free rate and position of double-J were assessed postoperatively using plain kidney-ureter-bladder X-film and non-contrast computerized tomography before removal of nephrostomy tube.

All patients were initially given empirical parenteral antibiotics, which included first to fourth generation cephalosporins, aminoglycosides, quinolones, monobactams, and penicillins upon admission. The parenteral antibiotics were shifted to appropriate ones according to the results of urine culture till the signs of infection subsided. Patients were prescribed oral Ketorolac 10mg three times per day to minimize urinary tract symptoms as needed, and allowed the use of sublingual buprenorphine 0.2mg on demand as needed. Overall dosages were documented and compared. Patients were randomized to receive emergency percutaneous nephrolithotomy or percutaneous nephrostomy according to a random numbers Table. The primary end point was the time taken until WBC normalization at 10.000/mm^3^ or less and a temperature of 37.4ºC or lower. The secondary end points were the comparison of analgesic consumption, length of stay, and related complications.

In the emergent percutaneous nephrolithotomy group, patients were placed in a prone position under endotracheal general anesthesia. All procedures were performed under sonographic guidance along the middle or upper calyx without retrograde ureteric catherization, and by percutaneous nephroscope (20.8Fr. Wolf) combined with 30Fr. Amplatz sheath, low pressure continuous normal saline irrigation, and the lithoclast (0.8mm probe, Swiss LithoClast®) to disintegrate the stones. The nephroscope ensued under direct vision after consecutive dilatation of the percutaneous nephrostomy tract. Simultaneously, lithotripsy was performed by hitting the stone’s center, breaking it into pieces as small as possible, and using the probe tip as the reference. When fragment size was deemed small enough, fragments were then retrieved from the uretero-pelvic junction under direct vision with a nephroscopic grasper. Surgery was concluded when no fragments remained in the entirety of the uretero-pelvic junction. Double-J ureteral stent and nephrostomy tube were placed routinely and double-J ureteral stent was left indwelling for two weeks. All procedures were performed by the same urologist to ensure uniform skill and experience level. Operation time was recorded starting from the insertion of percutaneous nephrostomy puncture needles until the placement of the nephrostomy tube.

In the emergency percutaneous nephrostomy group, emergency percutaneous nephrostomy (14Fr. nephrostomy tube) was performed in the angiography suite by a board-certified interventional radiologist using sonographic guidance with the patient under local anesthesia. Elective percutaneous nephrolithotomy was performed within 72 hours of diagnosis if the patient was hemodynamically stable (blood pressure of more than 110/60mmHg, heart rate of no more than 90 beats per minute, respiratory rate of no more than 20 breaths per minute and renal function within normal limits) after the initial parenteral antibiotics treatment.

All the enrolled patients were discharged after confirmation of double-J ureteral stent in situ and disappearance of all signs of infection (WBC normalization less than 8.000/mm3 and body temperature lower than 37.4ºC).

Statistical analysis was performed using SPSS® version 14.0.1. The Mann-Whitney U test, chi-square test, and Fisher’s exact test were all used as appropriate. P-values lower than 0.05 were considered significant.

## RESULTS

**Figure 1:**
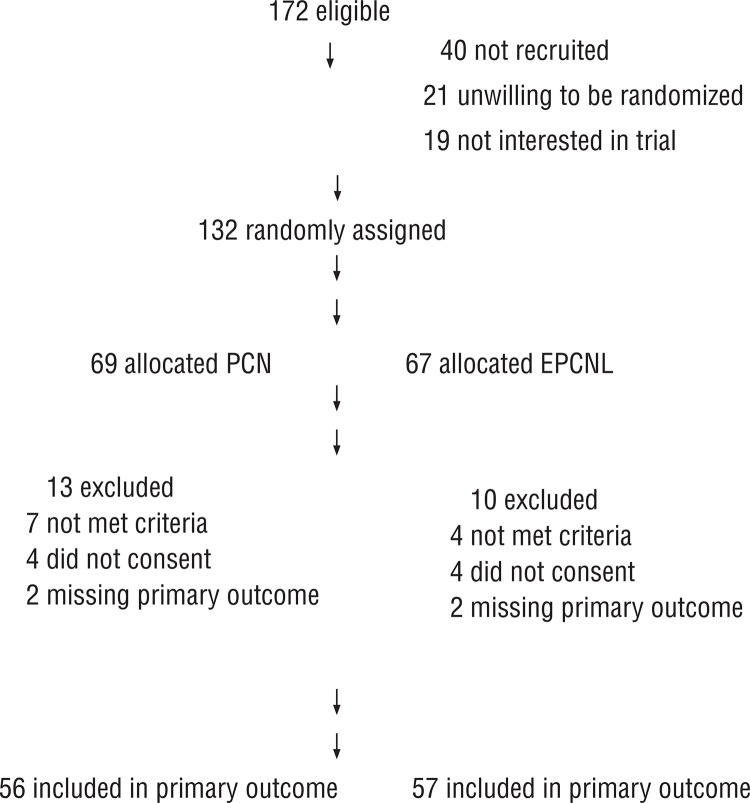
Summary of study disposition.

A total of 172 patients were eligible and prospectively randomized into two groups before they entered the operation room. In the percutaneous nephrostomy group, a total of 69 patients were available for consideration. Among the 69 patients, 7 did not meet the inclusion criteria with stable hemodynamic status and proper organ perfusions, and an additional 4 refused to sign the consent forms and were removed from the study. In all, a total of 58 patients were enrolled and received emergency percutaneous nephrostomy. Elective percutaneous nephrolithotomy treatment within 72 hours of diagnosis was made available if patients were deemed hemodynamically stable after their initial parenteral antibiotics treatment. In the emergency percutaneous nephrolithotomy group, a total of 67 patients were available. Among the 67 patients, 4 did not meet the inclusion criteria with stable hemodynamic status and proper organ perfusions, and an additional 4 refused to sign the consent forms and were removed from the study. In all, a total of 59 patients were enrolled and received emergency percutaneous nephrostomy. In both groups, there were 2 patients who were eventually unable to receive their allocation of treatment due to an inability to follow-up post-randomization. Thus, analysis was done with 56 and 57 patients as the denominator in each randomization arm (Figure-1).

Numbers of participants declining further follow-up or not responding are cumulative in direction of participant flow.

No significant statistical difference was observed in patient age, gender distribution, body mass index, stone size, stone burden, stone composition, stone laterality, operation times, or infected organisms (Table-1).

**Table 1:** Patients Demographics and Perioperative Data.

Characteristic	PCN group	EPCNL group	P value
	N=56	N=57	
**Age (year)** ^**a**^			**0.462**
Mean	58.91±11.18	58.12±12.53	
Range	33-76	39-89	
**Gender** ^**b**^			**0.751**
Male	36 (64.29)	35 (61.40)	
Female	20 (35.71)	22 (38.60)	
Body mass index ^**a**^	25.49±2.69	25.13±2.79	0.503
Male ^a^	25.54±2.72	25.46±2.38	0.958
Female ^a^	25.41±2.70	24.60±3.33	0.266
**Stone sizes(mm)**			
Length(mm) ^a^	24.88±2.79	25.47±3.80	0.901
Width(mm) ^a^	15.04±4.23	14.40±2.61	0.815
**Stone burden** ^**a**^	376.68±127.34	366.46±81.98	**0.654**
**Laterality** ^**b**^			**0.774**
Right	30 (53.57)	29 (50.88)	
Left	26 (46.43)	28 (49.12)	
Operative times (mins) ^a^	33.43±6.13	33.96±.31	0.647
**Culture organisms** ^**c**^			**0.997**
Escherichia coli	18 (32.14)	17 (29.82)	
Proteus mirabilis	8 (14.29)	9 (15.79)	
Pseudomonas aeruginosa	8 (14.29)	9 (15.79)	
Staphylococcus aureus	4 (7.14)	6 (10.53)	
**β-Hemolytic Streptococcus species**	2 (3.57)	1 (1.75)	
Candida albicans	2 (3.57)	2 (351)	
Multiple organisms	2 (3.57)	3 (5.26)	
Negative cultures	12 (21.43)	10 (17.54)	
**Appearance of kidney urine**			**0.886**
Cloudy	2 (3.57)	3 (5.26)	
Turbid	23 (41.07)	24 (42.11)	
Blood-stained	12 (21.43)	13 (22.81)	
Purulent	19 (33.93)	18 (31.58)	

Values are presented as mean±standard deviation or number (%); ^a^ Mann-Whitney U test; ^b^ Chi-square test; ^c^ Fisher’s exact test

The length of hospital stays (in days) was 10.09±3.43 for the emergency percutaneous nephrostomy group and 8.18±2.72 for the percutaneous nephrolithotomy group. This set of data noted a significant difference between groups (Table-2). There was no difference observed between groups with regard to white blood cell count (mm^3^), time to normalization of white blood cell count (in days), body temperature (in ºC), time to normalization of body temperature (in days), C-reactive proteins (in mg/dL), time taken for C-reactive proteins to decrease over 25% (in days), procalcitonin (in ng/mL), or complication rates (thrombocytopenia) (Table-2). However, analgesic consumptions were 30.89±10.83 in the emergency percutaneous nephrostomy group and 39.82±14.45 in the percutaneous nephrolithotomy group, with a significant difference. No patients suffered from postoperative exacerbation of the clinical condition and there were no postoperative mortalities in our study. All the uretero-pelvic junction stones were evacuated completely. The status of stone free was defined as total absence of residual stones and confirmed by non-contrast computerized tomography.

**Table 2:** Surgical Results and Complications.

	PCN group	EPCNL group	P value ^a^
Length of hospital stay(days)	10.09±3.43	8.18±2.72	0.001**
Respiration rate(time/min)	27.65±21.28	28.01±21.36	087
Pulse rate(beats/min)	94.24±22.57	95.03±23.04	0912
White blood count (mm^3^)	21760.71±7137.20	21420.68±5730.93	0.968
Time to normalization of White blood count (days)	4.89±1.71	4.30±1.46	0.062
Body temperature (ºC)	39.59±0.85	39.61±0.80	0.689
Time to normalization of body temperature (days)	2.63±1.38	2.49±1.44	0.438
C-reactive protein (mg/dL)	66.22±26.49	64.11±27.43	0.520
C-reactive protein decreased over 25% (days)	3.11±1.09	3.37±1.05	0.159
Procalcitonin (ng/mL)	26.98±20.78	25.89±28.72	0.240
Ketorolac (mg)	30.89±10.83	39.82±14.45	0.001**
Buprenorphine dosage (mg)	0.26±0.80	0.08±0.15	0.013*
**Complications**			
Thrombocytopenia ^b^	8 (14.29)	6 (10.53)	0.544

Values are presented as mean±standard deviation or number (%).

*p<0.05; **p<0.01

^a^ Mann-Whitney U test; ^b^ Chi-square test

## DISCUSSION

According to the European Association of Urology Guidelines on Urolithiasis (4), a large, obstructive renal stone with all signs of urinary tract infection is an urological emergency. Urgent decompression is often necessary to prevent further complications in infected kidneys presenting with hydronephrosis, secondary to stone-induced, unilateral, or bilateral renal obstructions. Currently, two options exist for urgent decompression of obstructed collecting systems: placement of an indwelling ureteral stent, or percutaneous placement of a nephrostomy tube. For decompression of the renal collecting system, ureteral stents and percutaneous nephrostomy catheters are equally effective. It is recommended that for sepsis presenting with obstructive stones, it is urgent for the collecting system to be decompressed, using either percutaneous drainage or ureteral stenting. Definitive treatment of the stone should be delayed until sepsis is resolved.

We conducted this randomized trial in order to evaluate the efficacy, related complications, and convalescence of emergency percutaneous nephrolithotomy when compared to percutaneous nephrostomy for decompression of the collecting system in cases of sepsis associated with large uretero-pelvic junction stone impaction. In our study, the inclusion criteria were broad enough to encompass cases with positive and negative cultures. Blood cultures may not always return positive for septicemia due to a variety of factors including fastidious organisms, prior antimicrobial therapy, growth inhibitory factors in the blood, and sampling error. Emergency percutaneous nephrolithotomy did not increase the incidence of complication rates (10.53%), and was lower when compared with the 14.29% incidence rate of percutaneous nephrostomy. The length of hospital stay was notably lower in the emergency percutaneous nephrolithotomy group. On the other hand, consumption of analgesics was notably lower in the emergency percutaneous nephrostomy group. As for the clinical normalization of index parameters (time until normalization of white blood cell count, body temperature, time until normalization of body temperature, C-reactive protein decrease of over 25%, and procalcitonin), there was no significant difference observed between the groups. It can be concluded, therefore, that emergency percutaneous nephrolithotomy neither leads to increased bacteremia nor is it significantly more hazardous when dealing with issues of acute obstruction. Besides, the superiority of emergency PCNL over emergency percutaneous nephrostomy includes obviation of multiple procedures, morbidities associated with ureteral stents or nephrostomy tubes, risk associated with drainage procedure, etc.

Traditionally, percutaneous nephrolithotomy has been contraindicated in unstable patients with sepsis because internal instrumentation is not advocated for such patients. Percutaneous nephrolithotomy, on the other hand, may be contraindicated or should be performed with extra care in patients presenting with bleeding diathesis (disseminated intravascular coagulopathy, severe thrombocytopenia, or prolonged prothrombin and partial thromboplastin times), cardiopulmonary insufficiency resulting in aggravation of respiratory symptoms when placed in a prone position, severe spinal dysraphism, and other causes of an abnormal body habitus, ectopic kidneys, and retrorenal colon. Lee et al. reports that 65 of 69 (94.2%) patients with urosepsis improved dramatically following percutaneous drainage (6). In our sample, all patients treated with percutaneous nephrolithotomy improved postoperatively. Lang and Price report a mortality rate of 8% after emergency percutaneous nephrostomy and 12% for surgical treatment of urosepsis secondary to obstruction (7). Even though a direct comparison cannot be made because their study was performed 30 years ago, there were no deaths after percutaneous nephrolithotomy in our study.

Fortunately, all the uretero-pelvic junction stones were evacuated completely. The status of stone free was achieved and confirmed by non-contrast computerized tomography. Although we achieved positive results with emergency percutaneous nephrolithotomy for obstructive uretero-pelvic junction stones, our study limitations involved the exclusion of patients with a single uretero-pelvic junction stone combined with multiple renal stones, uncorrected coagulopathy, and unstable hemodynamic sepsis. Emergency percutaneous nephrolithotomy is still contraindicated in bleeding diathesis, tumor in the presumptive access tract area, potential malignant kidney tumor, and pregnancy. Low risk patients with initial favorable response to treatment is the group to offer emergency PCNL.

Systemic inflammatory response syndrome (SIRS) defines a clinical response to a nonspecific insult of either infectious or noninfectious origin. SIRS is determined in the presence of two or more of the following variables: an elevated temperature over 38.0°C, a subnormal temperature below 36.0 ºC, a heart rate greater than 90 beats per minute, a respiratory rate greater than 20 breaths per minute, PaCO2 below 32 Torr, a white blood cell count over 12.000/mm^3^ or under 4.000/mm^3^, or over 10% immature (band form) forms (8-14). Sepsis is the systemic response to infection and is defined as the presence of SIRS in addition to a documented or presumed infection.

## CONCLUSIONS

This study confirms that emergency percutaneous nephrolithotomy may be as safe as early percutaneous nephrolithotomy in a selected low risk patients with sepsis-associated large, obstructive stone.

## ABBREVIATIONS

PCN: Percutaneous Nephrostomy
PCN: Percutaneous Nephrolithotomy
CT: computed tomography
UTI: urinary tract infections
